# Substance P enhances lactic acid and tyramine production in *Enterococcus faecalis* V583 and promotes its cytotoxic effect on intestinal Caco-2/TC7 cells

**DOI:** 10.1186/s13099-017-0171-3

**Published:** 2017-04-21

**Authors:** Kelly Biaggini, Valérie Borrel, Sabine Szunerits, Rabah Boukherroub, Awa N’Diaye, Arthur Zébré, Maryse Bonnin-Jusserand, Guillaume Duflos, Marc Feuilloley, Djamel Drider, Pierre Déchelotte, Nathalie Connil

**Affiliations:** 1 Laboratoire de Microbiologie, Signaux et Microenvironnement (EA4312), Université de Rouen/IUT d’Evreux, 55, rue saint Germain, 27000 Evreux, France; 2 Institute of Electronics, Microelectronics and Nanotechnology, UMR-CNRS 8520, Université Lille 1, Villeneuve d’Ascq, France; 3 Institut Charles Viollette, EA7394, Université du Littoral Côte d’Opale, Boulogne Sur Mer, France; 4 Laboratoire de Sécurité des Aliments, Département des Produits de la Pêche et de l’Aquaculture, ANSES, Boulogne Sur Mer, France; 5 Institut Charles Viollette, EA7394, Université Lille 1 – Sciences et Technologies, Villeneuve d’Ascq, France; 6 INSERM Unité 1073 «Nutrition, Inflammation et dysfonction de l’axe intestin-cerveau», Université de Rouen, Rouen, France

## Abstract

**Background:**

*Enterococcus faecalis*, generally considered as a saprophytic bowel commensal, has recently emerged as an important nosocomial pathogen causing severe urinary tract infections, surgical wound infections, bacteremia, and bacterial endocarditis. This bacterium is capable of forming biofilms on various surfaces and its high level of antibiotic resistance contributes to its pathogenicity. The aim of this study was to evaluate the effect on *E. faecalis,* of Substance P (SP), an antimicrobial peptide that is produced in the gut and skin.

**Results:**

We found that SP did not have antibacterial activity against *E. faecalis* V583 (MIC >1000 µg/ml). Conversely, SP stimulated aggregation, hydrophobicity, lactic acid and tyramine production in this bacterium. The cytotoxicity and bacterial translocation were also accelerated when *E. faecalis* V583 were pretreated with SP before infection of intestinal Caco-2/TC7 cells.

**Conclusion:**

SP can modulate the physiology of *E. faecalis*. Extensive studies are now needed to screen within the human microbiota which bacteria are responsive to host molecules, and to identify their sensors.

## Background

Antimicrobial peptides are produced by many living cells in animals and plants, and constitute integral components of innate host defense [1–3]. Several neuropeptides also have antimicrobial activity such as neuropeptide Y (NPY) and Substance P (SP) [4–7], and are known to be implicated in the microbiota–gut–brain axis [8]. SP is an undecapeptide of the tachykinin family which is abundant in the skin [9] and the gut [10]. In the skin, SP is considered as a major mediator of inflammation. It contributes to the pathogenesis of numerous skin diseases, like psoriasis, atopic dermatitis and acne, and can modulate the virulence of *Bacillus cereus*, *Staphylococcus aureus* and *Staphylococcus epidermidis* [9, 11]. In the bowel, SP is expressed in both myenteric and submucosal plexuses and is found within intrinsic and extrinsic sensory neurons. Immune effector cells, such as monocytes, macrophages, eosinophils, and lymphocytes, also express SP. A role of various levels of SP in the pathophysiology of inflammatory bowel disease has been suggested but disparate results were reported depending of the studies [10].

*Enterococcus faecalis* is a common resident of the gastrointestinal tract of humans and animals, and a wide-spread Gram-positive lactic acid bacterium that can be isolated from a variety of habitats, including fermented foods, human milk and vaginal secretions. *E. faecalis* Symbioflor 1 is also used as probiotic for more than 20 years and has been reported to reduce the number of relapses in patients with chronic recurrent hypertrophic sinusitis, as well as the number and severity of relapses in patients with chronic recurrent bronchitis [12]. Nevertheless, the use of enterococci as a probiotic or cheese starter remains controversial, due to biogenic amines production (tyramine and putrescine) that are toxic for human health [13, 14] and the risks of transfer of antimicrobial resistance and virulence genes to human strains [15, 16]. In fact, some strains, such as *E. faecalis* V583, are known to be opportunistic pathogen. This bacterium originates from a patient suffering from a persistent bloodstream infection, and it can cause diseases like urinary tract infections, bacteremia, and infective endocarditis in immunocompromised patients [17, 18]. These infections may be problematic because some *E. faecalis* strains are resistant to many antibiotics including vancomycin, and Enterococci are now considered among the most prevalent nosocomial pathogens [19, 20]. These multidrug-resistant (MDR) enterococci may colonize the patient after perturbation of the native flora by antibiotic treatment. Indeed, a recent study showed that commensal strains of *E. faecalis* generally protect the gut by producing pheromone peptides that can kill MDR *E. faecalis* V583 [21].

The opportunistic pathogen *E. faecalis* has extraordinary capacities to grow under adverse conditions and to colonize and survive in a large range of ecological niches including in macrophages [22–24]. The mechanism by which this bacterium is able to cross the barrier from a commensal to become a pathobiont may be a thin line [25] and is not well understood. In the host or after infection, *E. faecalis* may be in contact with various eukaryotic molecules that have antimicrobial properties and help fighting against the pathogen or in the contrary can contribute to promote the bacterial colonization. Therefore, the aim of this study was to determine the effect of SP on *E. faecalis* V583.

## Results

### MIC analysis and microscopic observations

**Fig. 1:**
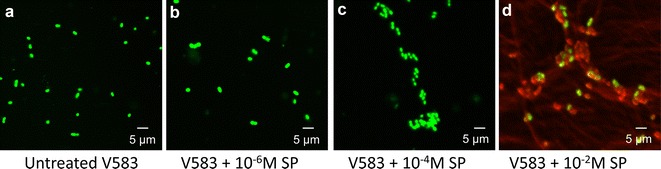
LIVE/DEAD analysis of *E. faecalis* V583 by confocal microscopy after 2 h exposure to SP. Viable (green bacteria) stained with SYTO 9, dead (red) bacteria stained with PI. **a** Untreated V583, **b** V583 + 10^−6^ M SP, **c** V583 + 10^−4^ M SP, **d** V583 + 10^−2^ M SP. *Scale bar* 5 µm

**Fig. 2:**
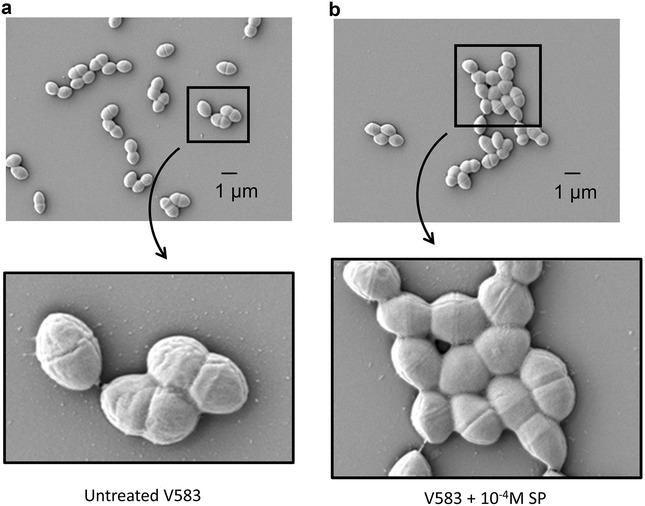
Scanning electron microscopic (SEM) images of *E. faecalis* V583 after 2 h exposure to SP. **a** Untreated V583, **b** V583 + 10^−4^ M SP. *Scale bar* 1 µm

No MIC value could be obtained using the microdilution method in Mueller–Hinton (MH) broth. Turbidity was seen in all the microplate wells (MIC >1000 µg/ml). Thus, antimicrobial effect of SP on *E. faecalis* V583 was also studied using confocal analysis and the LIVE/DEAD BacLight Bacterial Viability Assay Kit. It can be clearly seen from Fig. 1a that untreated control *E. faecalis* V583 appeared green (indicating viable cells stained by SYTO 9). When the bacteria were exposed for 2 h with 10^−6^ M (Fig. 1b) or 10^−4^ M SP (Fig. 1c), no additional dead cells were detected. Unexpectedly, a treatment with 10^−2^ M SP led to approximately 50% of red bacteria demonstrating dead cells stained with propidium iodide (PI) (Fig. 1d), although MIC analysis had not shown antimicrobial activity even for high concentrations of SP. Moreover, confocal observations showed that bacteria seem to slightly agglutinate when exposed to 10^−4^ or 10^−2^ M SP compared to untreated bacteria and bacteria exposed to 10^−6^ M. SEM analysis confirmed these observations. Untreated *E. faecalis* V583 (Fig. 2a) were mostly associated by 2 or 4 cells, whereas bacteria exposed to 10^−4^ M SP (Fig. 2b) can appear in cluster of more than 10 cells.

### Hydrophobicity

**Fig. 3:**
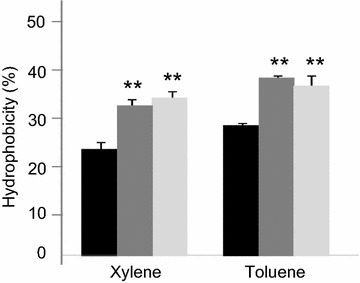
Cell surface hydrophobicity of *E. faecalis* V583 determined by a MATS test after 2 h exposure to SP. *Black* untreated bacteria, *dark grey E. faecalis* V583 exposed to 10^−6^ M SP, *light grey E. faecalis* V583 exposed to 10^−4^ M SP, **P < 0.01 versus untreated bacteria. Results are the mean ± SEM of at least three independent experiments

Aggregation of bacteria is often associated with hydrophobicity, therefore, the hydrophobicity of *E. faecalis* V583 treated or not with SP was then studied. The results are presented in Fig. 3. As expected, when the bacteria were exposed to SP, hydrophobicity slightly increased.

### Lactic acid and tyramine quantification

**Fig. 4:**
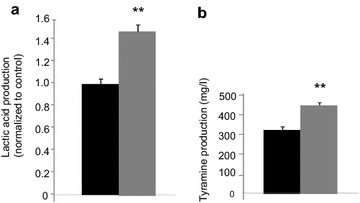
**a** Acid lactic and **b** tyramine production by *E. faecalis* V583 exposed to SP. *Black* untreated bacteria, *grey E. faecalis* V583 exposed to 10^−6^ M SP, **P < 0.01 versus untreated bacteria. Results are the mean ± SEM of at least three independent experiments

Lactic acid metabolism has been linked to virulence in *E. faecalis* [23] so this parameter was quantified. Figure 4a shows the lactic acid production normalized to control. Exposure of *E. faecalis* V583 to SP led to an increase of lactic acid production but the pH was not modified despite the acid production (data not shown). Tyramine was also overproduced when the bacteria were treated with SP (Fig. 4b). This may explain the absence of acidification.

In order to evaluate the pathogenicity of *E. faecalis* V583 after treatment with SP, in vitro assay were then conducted using Caco-2/TC7 intestinal epithelial cells.

### Cytotoxicity and quantification of IL-8

**Fig. 5:**
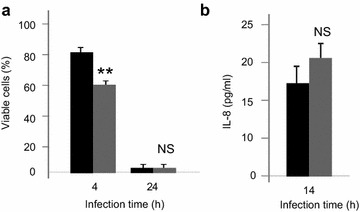
**a** Viability of Caco-2/TC7 cells and **b** IL-8 secretion after infection with *E. faecalis* V583 exposed to SP. *Black* infection with untreated bacteria, *grey* infection with *E. faecalis* V583 exposed to 10^−6^ M SP, *P < 0.05 versus untreated bacteria. Results are the mean ± SEM of at least three independent experiments

The cytotoxicity was determined by measuring the remaining viable cells with NR assay (Fig. 5a). The results show that Caco-2/TC7 cells were killed more rapidly when infected with *E. faecalis* V583 pre-exposed to 10^−6^ M SP. Indeed, after 4 h of infection, 80 ± 2% of the cells co-incubated with *E. faecalis* V583 remained viable, whereas only 61 ± 1% if the bacteria were treated with SP before the infection. Quantification of IL-8 (Fig. 5b) show no significant effect of SP on the proinflammatory potential of *E. faecalis* V583.

### Transepithelial electrical resistance (TER) and bacterial translocation

**Fig. 6:**
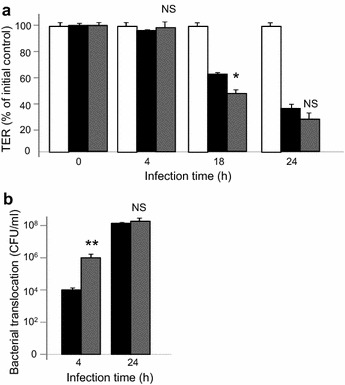
**a** Transepithelial resistance (TER) of Caco-2/TC7 cells. *White* TER of non infected cells, *black* TER of cells infected with untreated bacteria, *grey* TER of cells infected with *E. faecalis* V583 pretreated with 10^−6^ M SP. **b** Bacterial translocation of *E. faecalis* V583 across Caco-2/TC7 cells. *black* untreated bacteria, *grey E. faecalis* V583 pretreated with 10^−6^ M SP. *NS* not significant, *P < 0.05 versus untreated bacteria. Results are the mean ± SEM of at least three independent experiments

The transepithelial resistance of differentiated Caco-2/TC7 was monitored during 24 h (Fig. 6a). The results show that TER decreased more rapidly at 18 h of infection if the bacteria were exposed to SP (−53 ± 1%) compared to infection with untreated bacteria (−38 ± 0.5%). After 24 h of infection, Caco-2/TC7 cells were damaged in both cases, there is no longer any statistically significant difference. Unexpectedly, the translocation assay show that even before the decrease in TER, *E. faecalis* V583 has moved from the apical to basolateral compartment and 100 times more bacteria exposed to SP crossed the epithelial barrier than untreated bacteria. After 24 h of infection, and due to the alteration of the monolayer (as this was previously seen by TER measurement), all the bacteria, exposed or not to SP were recovered in the basolateral compartment.

## Discussion

Neuropeptides such as SP are important transmitters in the bidirectional gut–brain communication network that may influence the activity of the gastrointestinal microbiota and its interaction with the gut-brain axis [8]. As SP is known to display antimicrobial activity against oral, respiratory, gut and skin bacteria [5, 26] but also to conversely enhance virulence of *Bacillus cereus*, *Staphylococcus aureus* [9] and *Pseudomonas fluorescens* [27], the aim of this study was to determine its effect on *E. faecalis* V583.

El Karim et al. found that *E. faecalis* NCTC 12697 is highly resistant to SP with a MIC >500 µg/ml [26]. Similarly, our MIC analysis showed no antibacterial activity of SP against *E. faecalis* V583 even for a high concentration of 1000 µg/ml (7 × 10^−4^ M). However, confocal microscopy using LIVE/DEAD BacLight Bacterial Viability Assay revealed some dead cells for SP >10^−4^ M and the bacteria seem to slightly agglutinate which was then confirmed by SEM microscopy.

SP is a cationic antimicrobial peptide that may kill bacteria by pore formation and/or probably also act as a metabolic inhibitor [4]. To a certain extent, in our study, aggregation of *E. faecalis* V583, may protect the bacteria from SP, but conversely in some cases aggregation/agglutination of bacteria is also known to be inherent of the mechanism of action of some antimicrobial peptides [28, 29]. In addition, aggregation of bacteria is often associated with hydrophobicity, and these two parameters can help to adhere to different surfaces including intestinal epithelial cells [30]. Therefore, *E. faecalis* V583 was examined for its hydrophobicity according to exposure to SP. The results showed that a treatment of the bacteria with SP increased the hydrophobicity value. This allows us to hypothesize that SP in the gastrointestinal tract may promote adhesion of *E. faecalis* and/or other gut microbes to the mucosa.

As *E. faecalis* V583 is known to be an opportunistic pathogen, we examined some virulence properties of the bacteria following SP exposure. We found that lactic acid production was enhanced when *E. faecalis* V583 is treated with SP. Lactic acid produced by the lactate dehydrogenase (LDH) enzyme, is implicated in multiple stress resistance and virulence in *E. faecalis* [23] and was also demonstrated to be a potential virulence factor in *Streptococcus* [31]. In our study, the increase of lactic acid production during exposure of the bacteria to SP didn’t result to a fall in pH. In fact, to protect the bacteria from its own acid production, *E. faecalis* is able to decarboxylate amino acid, especially tyrosine in tyramine [32–34]. Tyramine biosynthesis is transcriptionally induced at low pH [35, 36] and this improves the fitness of *E. faecalis* and *Enterococcus durans* in acidic environments such as in the gastrointestinal tract [37]. We showed that exposure to SP enhanced the production of tyramine by *E. faecalis* V583 concomitantly with lactic acid, this may explain why no modification in pH was observed in our experiment. The biosynthesis of tyramine is a general species trait of *E. faecalis* [13]. Large amounts of this biogenic amine can cause toxicological effects [14, 38] including migraines and hypertension and sometimes problems as serious as cerebral haemorrhages. Thus, production of tyramine by gut commensal *E. faecalis* exposed to SP deserves more attention.

Subsequently, the pathogenicity of *E. faecalis* V583 treated with SP was evaluated using Caco-2/TC7 intestinal epithelial cells. The results showed that the cytotoxicity of *E. faecalis* V583 was enhanced with SP exposure and the permeability of differentiated Caco-2/TC7 cells increased rapidly, allowing the bacteria to translocate faster to the basolateral compartment. Linares et al. recently found that tyramine is toxic for HT29 intestinal cell cultures and induces necrosis [14]. Thus, the increase of the cytotoxic effect of *E. faecalis* V583, pretreated with SP, on Caco-2/TC7 cells, could be partially attributed to the overproduction of tyramine we noticed before.

In previous works, we showed that SP can modulate the swarm and proinflammatory potential of *P. fluorescens* [27] and strongly stimulated the cytotoxicity of *B. cereus* [9], so this neuropeptide, abundant in the skin and the gut, may promote pathogenicity in various bacteria. In fact, microbial endocrinology shows that, through their long coexistence with animals and plants, microorganisms have evolved sensors for detecting eukaryotic molecules [39] and a bidirectional communication exists between the gut and its microflora [40, 41].

Ef-Tu is a moonlighting protein [42, 43] that has been found as the SP sensor in *Bacillus cereus* [9], and recently identified also as the SP-interacting protein in *S. aureus* and *S. epidermidis* [11]. The putative binding site of SP in *E. faecalis* is now under investigation in our laboratory and the preliminary results showed that Ef-Tu would indeed be the sensor. It remains also to determine which other bacteria within the gut microbiota expresses neuropeptide receptors or releases metabolites that are ligands for eukaryotic neuropeptide receptors. This may probably help to better understand the complex relationship between brain, gut and microbiota, and to found adequate therapy in intestinal disorders.

## Methods

### Bacterial strain, culture conditions and reagents

The human clinical isolate *E. faecalis* V583 was used in our study [18]. This strain was cultivated at 37 °C in Brain Heart Infusion (BHI) medium and stored at −80 °C in 50% (v/v) glycerol. SP was synthesized by PolyPeptide group. For each experiment, a stock solution of the peptide was freshly prepared in Milli-Q water and filter-sterilized.

### Minimal inhibitory concentration (MIC) analysis

MIC was studied by microdilution in Mueller–Hinton (MH) broth using an inoculum of 10^5^ colony forming units per ml. Microtiter plate containing triplicate twofold dilution series of 1000 µg/ml SP was incubated overnight at 37 °C. Turbidity was observed at the end of incubation.

### LIVE/DEAD analysis by confocal microscopy

Viability of *E. faecalis* V583 treated or not with SP was analyzed using the LIVE/DEAD *BacLight* Bacterial Viability Kit for microscopy (L7007, Invitrogen). In this system, live bacteria stain with SYTO 9 to produce a green fluorescence whereas dead bacteria stain with propidium iodide (PI) to produce a red fluorescence. Briefly, 3 µl of the SYTO 9/PI mixture, prepared according to the manufacturer’s instruction, was added to the bacterial cells previously treated with 10^−2^, 10^−4^ or 10^−6^ M of SP for 2 h, and untreated cells were kept as control. The samples were incubated for 15 min in dark at room temperature and 5 µl of this sample was trapped in between coverslip and glass slide. The slide was viewed under a confocal laser-scanning microscope (LSM 710 CLSM, Zeiss), using 60× objective sequentially using fluorescence setting for FITC (green/SYTO 9, viable cells) and PI (red/PI, dead cells) filters, respectively, followed by phase contrast and bright field settings. SYTO 9 and PI images were merged and acquired using ZEN^®^ software (Zeiss).

### Scanning electron microscopy (SEM)

10^8^ CFU/ml bacterial suspension was incubated with 10^−4^ M of SP in phosphate buffered saline (PBS) for 2 h. At the end of incubation, the bacteria were washed with PBS and fixed on coverslips with 1% glutaraldehyde in 0.1 M sodium cacodylate buffer (pH 7.3) for 2 h. After washing with Milli-Q water, bacteria were dehydrated with increasing ethanol concentration (50, 70, 95 and 100%). Dry coverslips were mounted on stubs and coated with 5 nm platinum (Quorum Technologies Q150T, Elexience, France). Bacteria were observed with a secondary detector in a Zeiss SEM Merlin Compact VP (Zeiss, France) operating at 5 kV.

### Hydrophobicity

The hydrophobicity of *E. faecalis* was evaluated by the microbial adhesion to solvent (MATS) test, according to Al Atya et al. [44] and Kos et al. [45] with a slight modification. Bacteria grown in BHI with or without SP (10^−4^ or 10^−6^ M) for 18 h at 37 °C, were harvested by centrifugation at 5000*g* for 15 min, washed twice, and resuspended in PBS to approximately 10^8^ CFU/ml. The absorbance of the cell suspension was measured at 600 nm (*A*_0_). One ml of solvent was added to 3 ml of cell suspension. After a 10 min preincubation at room temperature, the two-phase system was mixed by vortexing for 2 min. The aqueous phase was removed after 20 min of incubation at room temperature, and the absorbance at 600 nm (*A*_1_) was measured. Two apolar solvents were tested: xylene and toluene. Bacterial adhesion to these solvents reflects cell surface hydrophobicity. The percentage of bacterial adhesion to the solvents was calculated as 1 − (*A*_1_/*A*_0_) × 100.

### Lactic acid and tyramine quantification

Lactic acid was quantified as recently described [44]. Briefly, *E. faecalis* were grown in BHI with or without 10^−6^ M SP at 37 °C, and samples were withdrawn after 14 h of incubation, centrifuged (10,000*g*, 4 °C, 10 min), and sterilized by filtration using Millipore filter (0.2 µm). The concentration of lactic acid in the samples was determined by HPLC spectra system P1000XR (Thermo, USA).

For tyramine analysis, the bacterial samples were prepared similarly, except that tyrosine (800 mg/l) was added in the BHI medium [34]. The quantification was performed by mixing 20 µl of bacterial samples with 80 µl of perchloric acid (0.2 M) and 5 µl of 1.7 diaminoheptane (6.4 mg/ml), shaking until complete homogenization and centrifuging at 8000*g*, 4 °C, 5 min. The steps of derivatization using dansyl chloride and purification are described in Duflos et al. [46]. Hitachi Elite LaChrom HPLC System was used to quantify tyramine with Kromasil column (C18, 5 µm 100 Å; 25 cm × 4.6 mm).

### Caco-2/TC7 cells and culture

The eukaryotic cells used in our study were the intestinal Caco-2/TC7 cell line, a late passage of Caco-2 (P-198), which has been previously recommended for its performance and reproducibility [47]. Cells were routinely grown at 37 °C in 5% CO_2_–95% air atmosphere in Dulbecco’s Modified Eagle’s Medium (DMEM) supplemented with 15% heat-inactivated foetal calf serum, and Penicillin/Streptomycin. For experimental assays, the cells were seeded in 24-well tissue culture plates (for cytotoxicity assay and interleukine-8 (IL-8) quantification) or on insert (3 µm pore size) for 21 days to ensure epithelial differentiation (for transepithelial electrical resistance (TER) measurement and translocation assay). At the end of differentiation, the mean TEER value measured for control cells was approximately 700 Ω/cm^2^.

### Bacterial treatment with SP and infection of Caco-2/TC7 cells

For all the following experiments conducted on Caco-2/TC7 cells, only a dose of 10^−6^ M SP was used, which is more relevant to the physiological concentration in the gut. SP was added to the cultures of *E. faecalis* V583 at the beginning of the exponential growth phase. The bacteria were exposed for 2 h with the peptide, then centrifuged for 5 min at 8000*g*, washed and resuspended in cell culture medium without antibiotics and serum. Caco-2/TC7 cells were infected at a MOI (multiplicity of infection) of 100:1 with the bacteria treated or not with SP, and incubated for 15 h at 37 °C, CO_2_ 5%, unless otherwise stated.

### Cytotoxicity and quantification of IL-8

The cytotoxicity of *E. faecalis* V583 treated or not with SP was estimated by enumeration of the remaining Caco-2/TC7 viable cells, 4 and 24 h after infection, using the neutral red (NR) uptake assay. Only viable cells will take up NR by active transport and incorporate the dye into lysosomes. Following infection, Caco-2/TC7 cells were washed once in PBS and incubated with NR (50 µg/ml) for 1 h at 37 °C, CO_2_ 5%. After incubation, the cells were rinsed once in PBS, and NR incorporated in viable cells was extracted with a solution of 50% (v/v) ethanol and 1% (v/v) acetic acid for 10 min before measuring optical density at 550 nm.

The level of IL-8 was quantified in the supernatant of Caco-2/TC7 cells, 14 h after infection, using the CXCL8 ELISA Quantikine kits (R&D systems) according to the manufacturer’s protocol.

### Transepithelial electrical resistance (TER)

The effect of *E. faecalis* V583, treated or not with SP, on the TER of Caco-2/TC7 monolayers was measured at 0, 4, 18 and 24 h, using the Millicell Electrical Resistance System. For each condition tested, the TER values were expressed as percentages of the initial level measured in the insert.

### Bacterial translocation

After 4 and 24 h infection of the Caco-2/TC7 inserts, aliquots of 100 µl of the basolateral compartment were collected and the number of bacteria that crossed the epithelial monolayers was determined by serial dilution and plating onto BHI agar.

### Statistical analysis

All the assays were performed from at least three independent replicates. G_RAPH_P_AD_ P_RISM_ software and Student’s t test were used to compare the data statistically.
